# Supplementary material for: Learning curve and surgical outcome of robotic assisted colorectal surgery with ERAS program
Source: Sci Rep. 2022 Nov 29;12:20566. doi: 10.1038/s41598-022-24665-w (PMC9709162; doi:10.1038/s41598-022-24665-w)
Supplement: Supplementary file 1 — Supplementary Information 1. [file 41598_2022_24665_MOESM1_ESM.zip › LSC Raw data-Ñ[▒K/088/H_ñw╝╨░O▒Kñσ.pdf]

| 正式摘要(電子簽章：000000195475799)<br>W55 / W55 ☐英文標題(注意:無英文姓名-請補病人基本檔!) | ☐【讀取】憑證 |
|---|---|

# 臺中榮民總醫院

## 出院病歷摘要

轉入號： 轉出號：

[redacted]

| | |
|---|---|
| **(8)**流水編號 | |
| **(9)**入院日期 | 109/07/22 (2020) 直腸外科 W55 - 285 |
| **(10)**轉科日期 | |
| **(11)**出院日期 | 109/07/27 (2020) 住院天數計 5 日 |

### 診斷

- 入院 :Malignant tumor of DS junction
- 出院 :Malignant tumor of DS junction, s/p laparoscopic left hemicolectomy on 20200723, cT3N0M0.

### 主訴

- Accidentally found sigmoid colon cancer by colonoscopy during health examination on 2020/7/1.

### 病史

- This 69 years old female patient denied special systemic disease. She recieved health examination on 2020/7/1. In colonoscope, sigmoid tumor was noted from anal verge 28cm which pathology report showed malignancy. She denied changed bowel habbit, body weight loss, or bloody stool. She came to our OPD for help. Thus, she was admitted for surgical intervention.

### 體檢發現

- ...................................
  - General appearance :
    - easy looking , conscious clear , without cardiopulmonary distress
    - Vital signs日期帶入date 體溫(BT) 脈搏(P) 呼吸(R) 血壓(BP) 身高(BH) 體重(BW) BMI
      20200722 35.8℃ 80次/分 16次/分 124/72mmHg 162.9cm 63.9kg 24.1
    - Vital signs: B.P. 124/72 mmHg , P.R. 80 /min , R.R. 16 /min , B.T. 35.8 ℃
  - HEENT :
    - Eyes: no anemic conjunctiva, no icteric sclera, normal EOM
    - ENT: no otorrhea, no rhinorrhea, no erythematous mucosa, no injected tonsil, no deformity
  - Neck: supple, freely movable, trachea midline,no deformity, no engorged jugular vein , no palpable thyroid or lymph node enlargement
  - Chest:
    - Inspection: Grossly normal, symmetric and fully expansion, no accessory muscle used, no operation scar, no spider angiomata, no gynecomastia
    - Palpation: no palpable mass or nodule, no subcutaneous emphysema, no tenderness
    - Ausculation: clear breathing sound
  - Heart:
    - Palpation: no thrill, no heave
    - Percussion: normal heart size
    - Ausculation: regular heart beat, normal S1, S2, no gallop, no murmur
  - Extremities: No deformity, full ranged of motion without limitation, no clubbing finger or nail cyanosis, no pitting edema, no ulcer or pigmentation, no obvious skin lesion, no varicose vein, no bone, joint or muscle tenderness

**CRS CONDITION**

  - Karnofsky performance status: (100) Normal, no complaints, no evidence of disease
  - PE of Abdomen:
  - CEA: 1.83 ng/ml
  - Imaging Studies:
  - Colonoscope report: sigmoid tumor was noted from anal verge 28cm
  - Figures:
    1. colon & rectum

### 手術日期及方法

- 手術日期及方法20200723 1. Laparoscopic left hemicolectomy with end-to-side anastomosis, endo-GIA 60/4.1*1, ILS 29mm, air leak test done, no leak, re-inforcement with silk: nil.
  2. Vessel ligation: high ligation of IMA root, IMV root
  3. Cut margin: proximal 9 cm, distal 6 cm.
  4. Reperitonealization: Nil.
  5. One 12Fr. Penrose drain at plevis.

### 住院治療經過

- After admission, laparoscopic left hemicolectomy was smoothly performed on 20200723. We stopped IVF, removed Foley and penrose drain on POD2(7/25). Epidural PCA was also stopped on 7/26. With improving appetite and oral intake noted, she is allowed to be discharged under relatively stable condition.

### 合併症

- nil

### 一般檢查

- 累積報告 :

| DATE | TIME | ALB | TP | BIL.T | BIL.D | ALKP | AST | ALT | LDH | CHOL | TG | HDL-C | LDL | UA | NH3 | AMY |
|---|---|---|---|---|---|---|---|---|---|---|---|---|---|---|---|---|
| 1090723 | 0919 | | 7.2 | | | | | | | 159 | 85 | | 96 | 4.0 | | |

| DATE | TIME | NA | K | CL | CA | BUN | CREAT | Mg | P | CRP | ESR | PCT | LIPASE | LACTATE |
|---|---|---|---|---|---|---|---|---|---|---|---|---|---|---|
| 1090723 | 0919 | | | | | | | | | 0.268 | | | | |

- 放射線報告 :檢驗項目 **:CT ABDOMEN-SURVEY** 報告日期 **:1090726**
  上 訴：

- Result:
  Finding:
  Imaging Report Form for Colorectal Carcinoma (AJCC 8th)
  1. Imaging Date: 20200721
     Imaging Modality, Imaging by
       CT scan
     Imaging Protocol: Post IV contrast CT scan of the whole abdomen with 600cc oral contrast.
  2. Tumor location / Size
     Location:
       Descending
     Size:
       Measurable: 4.6 cm (largest diameter)
  3. Tumor invasion
     T3: Tumor invades non-peritonealized pericolonic or perirectal tissues
  4. Regional nodal metastasis
     N1: Yes
       number of suspicious lymph node: 5
       location:
         Pericolic/perirectal
  5. Distant metastasis (In this study)
     No or Equivocal
  6. Other findings: (1) Presence of segmental eccentric wall thickening of descending colon, about 4.6cm in length, colon cancer may be compatible.
     (2) Slight fat stranding at pericolic region, pericolic invasion cannot be ruled out.
     (3) Several small nodules at pericolic region, in favor of regional lymphadenopathy.
     (4) A 0.8cm calcification at S8 of liver, in favor of calcified granuloma.
     (5) Pancreas, spleen, gall bladder and bil. kidneys are not unusual.
     (6) No significant retroperitoneal lymphadenopathy.
     (7) Slight subpleural infiltration at bil. lower lungs.
     (9) DJD change of T and L spine.
  Impression:
  Compatible with descending colon cancer with pericolic invasion and regional lymphadenopathy.

  檢驗項目 **:CHEST PA VIEW** 報告日期 **:1090725**
  主 訴：
  Result:
  Finding:
  1. No cardiomegaly.
  2. Mild scoliosis and severe spondylosis of T-L spine. S/P Total shoulder replacement, left.
  3. No active lung lesion.
  4. Otherwise, no other significant finding.
  Impression:
  As above description.

## 特殊檢查

## 病理報告

## 其它

## 出院時情況

- 3.改門診治療

## 出院指示

| 藥名 | 劑量 | 劑量單位 | 用法 | 頻次 | 數量 | 數量單位 | 現況 | 天數 | 單位 |
|---|---|---|---|---|---|---|---|---|---|
| Gaslan tab 40mg | 1 | TAB | PO | QID | 36 | TAB | TH | 9 | 天 |
| Ultracet tab | 1 | TAB | PO | Q6HPRN | 28 | TAB | TH | 7 | 天 |

## 出院計劃

- 門診日期
  - date on 2020/08/04
- 門診追蹤

主治醫師 2062F 蔣鋒帆 / 住院醫師 9889F 陳俊男

正式摘要**(**電子簽章：**000000195475799)**

W55 / W55

doctorRank:B
